# Tissue Trafficking Kinetics of Rhesus Macaque Natural Killer Cells Measured by Serial Intravascular Staining

**DOI:** 10.3389/fimmu.2021.772332

**Published:** 2022-01-11

**Authors:** Ryland D. Mortlock, Chuanfeng Wu, E. Lake Potter, Diana M. Abraham, David S. J. Allan, So Gun Hong, Mario Roederer, Cynthia E. Dunbar

**Affiliations:** ^1^Translational Stem Cell Biology Branch, National Heart, Lung, and Blood Institute, National Institutes of Health, Bethesda, MD, United States; ^2^Vaccine Research Center, National Institute of Allergy and Infectious Diseases, National Institutes of Health, Bethesda, MD, United States; ^3^Cellular and Molecular Therapeutics Branch, National Heart, Lung, and Blood Institute, National Institutes of Health, Bethesda, MD, United States

**Keywords:** NK cells, tissue trafficking, intravascular staining, rhesus macaque, peripheral blood, lymph nodes

## Abstract

The *in vivo* tissue distribution and trafficking patterns of natural killer (NK) cells remain understudied. Animal models can help bridge the gap, and rhesus macaque (RM) primates faithfully recapitulate key elements of human NK cell biology. Here, we profiled the tissue distribution and localization patterns of three NK cell subsets across various RM tissues. We utilized serial intravascular staining (SIVS) to investigate the tissue trafficking kinetics at steady state and during recovery from CD16 depletion. We found that at steady state, CD16+ NK cells were selectively retained in the vasculature while CD56+ NK cells had a shorter residence time in peripheral blood. We also found that different subsets of NK cells had distinct trafficking kinetics to and from the lymph node as well as other lymphoid and non-lymphoid tissues. Lastly, we found that following administration of CD16-depleting antibody, CD16+ NK cells and their putative precursors retained a high proportion of continuously circulating cells, suggesting that regeneration of the CD16 NK compartment may take place in peripheral blood or the perivascular compartments of tissues.

## Introduction

Natural killer (NK) cells are innate lymphoid cells which play critical roles in controlling viral infection and eliminating cancerous cells. NK cells use a variety of germline encoded activating and inhibitory receptors to directly lyse target cells, bypassing the need for somatic hypermutation to generate antigen specific receptors. There are two well-studied subsets of human NK cells: CD56^bright^ and CD56^dim^, defined based on their relative expression of neural cell adhesion molecule (NCAM1 or CD56) which is a glycoprotein of the immunoglobulin superfamily involved in cell adhesion. CD56^bright^ NK cells predominate in secondary lymphoid tissues such as the lymph nodes (1, 2) and display enhanced release of cytokines after stimulation (3). CD56^dim^ NK cells are the primary NK cell subset in circulation and display an enhanced ability to release cytotoxic granules to kill target cells (4). They are characterized by their expression of the activating Fc receptor CD16 which is involved in antibody dependent cellular cytotoxicity.

CD56^bright^ and CD56^dim^ NK cells have numerous transcriptional and protein expression differences (5), including in homing receptors, with CD56^bright^ NK cells expressing CCR7 and CD62L (L-selectin) while CD56^dim^ NK cells have higher expression of the chemokine receptors CX3CR1 and CXCR1 (6, 7). Human NK subsets also have distinct tissue localization patterns. In a recent study of human autopsy samples, NK cells were most abundant in peripheral blood (PB), bone marrow (BM), spleen, and lung, with primarily a mature CD56^dim^ phenotype, whereas lower frequencies of NK cells were found in tonsil, lymph node (LN), and gut, with predominantly a CD56^bright^ phenotype (8). In addition to showing tissue-specific distribution of NK cells, the authors demonstrated differences in NK cell function and inferred differences in developmental pathways across tissues which is in line with other studies of tissue-resident NK (trNK) (9–13) including in rhesus macaques (14, 15). Early studies identified a population of liver-resident CD49a+ DX5 (CD49b)- NK cells in mouse which, unlike conventional NK cells in the blood, were dependent on the transcription factor T-bet but not Eomes for development (16, 17). A corresponding population of liver-resident NK cells was identified in humans (18), and a future study identified a population of CD56^bright^ NK cells which were Eomes^high^ and T-bet^low^ but displayed evidence of liver residence including expression of CXCR6 and CD69 (19). This population of CD49a+ liver-resident NK cells has also been identified in rhesus macaque (15). CD49a+ CD69+ trNK cells have been identified in human lung (20, 21) and CD69 and CXCR6 have been used as markers of lymphoid trNK cells (22).

The classical NK cell development model suggests continuous differentiation of CD56+ NK cells into CD16+ NK cells (23, 24) supported by some *in vitro* (25, 26) as well as *in vivo* evidence in mice (25, 27). However, there are data challenging this model including *in vitro* studies (28), observations from patients with GATA2 mutations (29), and clonal tracking of human paroxysmal nocturnal hemoglobinuria patients (30).

Non-human primate models have been utilized extensively to study NK cell biology due to the close phylogenetic relationship between humans and primates and the ability to perform experimental manipulations in primate models. Rhesus macaque (RM) primates have three distinct subsets of NK cells: CD56+CD16- (CD56+), CD56-CD16- (DN), and CD56-CD16+ (CD16+). RM CD56+ and CD16+ NK cells closely resemble human CD56^bright^ and CD56^dim^ NK cells respectively at the receptor and transcriptional level (31, 32). The RM DN NK subset is less studied, perhaps because CD56- NK cells are very rare in humans except in the case of chronic viral infection (33). Clonal tracking studies in RM have shed insight on the life history and possible development relationships between NK cell subsets (34–36). After transplantation of RMs with genetically barcoded hematopoietic stem and progenitor cells (HSPCs), CD56+ NK cells showed stable clonal patterns long-term after transplantation with clonal compositions similar to myeloid, B and T-cell lineages. On the other hand, CD16+ NK cells exhibited marked oligoclonal expansions after transplantation, with clones waxing and waning over time and little clonal resemblance to CD56+ NK cells, suggesting peripheral renewal independent of ongoing production from HSPCs, analogous to T memory cells (35).

NK cell trafficking *in vivo* remains an understudied topic even though it is critical to understanding both the function of NK subsets and their possible developmental relationships. Recently, new technologies have been developed for *in vivo* tracking of leukocytes, including intravascular staining (IVas), which involves intravenous infusion of a fluorescently conjugated cell-type specific or pan-leukocyte antibody immediately before necropsy or tissue sampling, permitting the separation of intravascular cells, which are stained by the antibody, from tissue-localized (TL) cells which are not stained (37). Note that the TL compartment comprises true tissue-resident (TR) cells as well as potentially slowly migrating cells that have remained in the tissue for at least the time period of the experiment.

Potter et al. advanced this protocol by developing a method for serial intravascular staining (SIVS) in which non-human primates are given repeated injections of differently colored anti-CD45 antibodies (38). Antibodies are given at a sub-saturating dose with an *in vivo* half-life of 8.5 minutes; thus the labeling can be considered a pulse which instantly tags leukocytes in circulation at the time of infusion. The method was determined to accurately discriminate between intravascular and TL cells when given immediately prior to necropsy; all cells collected from blood stained positive while most cells collected from lymph node (LN) stained negative, with the small percentage of positive cells located closer to CD31+ blood vessels than negative cells. Lastly, the antibody was shown to be retained on the surface of leukocytes during migration into tissues. Therefore, administration of multiple SIVS infusions can allow reconstruction of tissue trafficking patterns and kinetics. This technique has already been used to study T cell infiltration into gut tissues during acute graft-versus-host disease in RM (39).

In the current study, we use SIVS to profile NK cells and NK subsets across various RM tissues to contribute to our understanding of NK localization and trafficking, with insights into the life histories of these cell populations and implications for their functions.

## Materials and Methods

### Animals

The animals used in the study were Indian-origin rhesus macaques (*Macaca mulatta*). All procedures were carried out in accordance with institutional and national guidelines and laws governing research in animals including the Animal Welfare Act. The animal protocols were reviewed and approved by the NIAID and NHLBI Animal Care and Use Committees. The National Institutes of Health (NIH) and Bioqual Inc. (where some animals were housed) are accredited by the Association for Assessment and Accreditation of Laboratory Animal Care and are in full compliance with the Animal Welfare Act and Public Health Service Policy on Humane Care and Use of Laboratory Animals. Sedation and euthanization followed previously described protocols (38).

### αCD45 Infusions and Tissue Processing

For SIVS infusions, purified anti-CD45 antibody (mouse IgG1κ) was developed in house and conjugated to the Alexa Fluor dyes (Thermo Fisher) shown in **Figure 2A** using N-hydroxysuccinimide (NHS) chemistry. Conjugated antibodies were diluted in sterile Hanks’ buffered salt solution. For all infusions, 5 mL of antibody solution was administered over 30 seconds *via* a catheter placed in the saphenous vein. Animals DGA0 and DGF5 received antibodies at doses of 10 and 100 ug/kg respectively, and animals G45T, ZJ31, HAXR, TID, and K628 received antibodies at a dose of 30 ug/kg. All infusion reagents were tested for endotoxin and were found to be negative. Blood draws were taken from the femoral artery, and lymph node biopsies were taken from the specified sites (axillary, mesenteric, or inguinal).

PBMCs isolation was performed using Ficoll-Paque PLUS (GE Healthcare) gradient separation and standard procedures. Lymph nodes and spleen tissues were mechanically disrupted and filtered through a 70-μm cell strainer. For spleen, the single-cell suspension was enriched for lymphocytes using Ficoll-Paque PLUS gradient separation. Lung tissue was processed using gentleMACS C Tubes and gentleMACS Dissociator (Miltenyi Biotec) and digested using type I collagenase (Gibco) and deoxyribonuclease (DNase) I (Roche) shaking at 37°C for 1 hour. Red blood lysis was performed with diluted 10× RBC Lysis Buffer (BioLegend). Cells were washed with phosphate buffered saline (PBS) and filtered through a 70-μm cell strainer. Jejunum and liver tissues were digested with the collagenase/DNase solution for 1-hour shaking at 37°C and filtered through 70-μm filters. Cells were either washed and immediately analyzed or were cryopreserved in 10% dimethyl sulfoxide (DMSO) in fetal bovine serum (FBS) and stored in liquid nitrogen.

### Cell Staining and Flow Cytometry

Cryopreserved cells were thawed for staining and flow cytometric analysis. Cells were split into technical replicates and stained separately with antibody panel #1 and panel #2 (**Table S1**). For panel #1, intracellular Ki-67 staining was performed after surface markers staining using the intracellular staining protocol from the eBioscience Foxp3/Transcription Factor Staining kit. Flow cytometry was performed using the BD LSRFortessa™ Cell Analyzer.

### CD16 Depletion

The mouse anti-human CD16 monoclonal NK depleting antibody (clone 3G8) was obtained from the Nonhuman Primate Reagent Resource (http://www.nhpreagents.org) and used for NK depletion as described in previous studies (40). Animals HAXR and K628 in this study received a single 50 mg/kg intravenous dose of anti-CD16 depleting antibody as detailed in **Figure 5A**. The gating scheme utilized to follow CD16 depletion and technical validation for SIVS staining is shown in **Figure S7**.

### Data Analysis and Statistics

All flow cytometry data was analyzed using FlowJo v10. The SIVS proportions for T cells and each NK cell subset were exported and analyzed using R v4.0.1 (41). The two different antibody panels were used as technical replicates for quantifying SIVS proportions. The *networkD3* package v0.4 (42) was used to create Sankey diagrams such as in **Figure 2B**. Wilcoxon tests were performed between NK cell subsets using the *ggpubr* package v0.4.0 (43) with each animal as a biological replicate. Other packages used included *ggplot2*, *dplyr*, *plyr*, *reshape2*, and *RColorBrewer*. The line plot in **Figure 5C** was created using GraphPad Prism v8 (GraphPad Software).

## Results

### NK Cell Proportion and Subset Distribution Varies Across Tissue Sites in Rhesus Macaques

**Figure 1:**
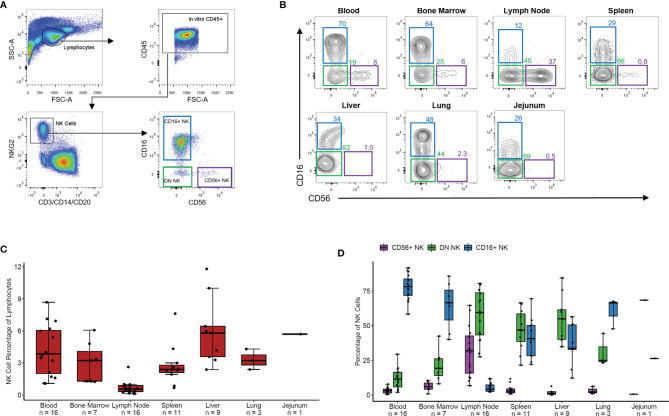
NK phenotypes in rhesus blood and tissues. **(A)** Representative gating scheme of rhesus macaque NK cells defined as CD3- CD20- CD14- NKG2+ followed by delineation of CD56+, CD56-CD16- (DN), and CD16+ NK cell subsets. **(B)** Representative flow plots of NK cell subsets in tissues. **(C)** Boxplot of the NK cell percentage of total lymphocyte for the seven tissues shown in panel **(B)**. **(D)** Boxplot showing the percentage of each NK cell subset of the total NK cell population for the seven tissues shown in panels **(B, C)** For boxplots, each data point within a tissue is from a different animal. Box shows interquartile range and black line shows median value. Whiskers extend to the largest and smallest values within 1.5 times the interquartile range.

We measured the overall frequency of NK cells within lymphocytes and of CD56+, DN, and CD16+ NK cell subsets within the NK cell population in blood and six tissues from 20 healthy RMs. NK cells were defined as negative for the lineage markers CD3, CD14, and CD20 and positive for the NK cell marker NKG2 *via* an antibody which binds to both NKG2A and NKG2C, surface receptors present on mature NK cells in non-human primates (14). Representative gating is shown in **Figure 1A**. Three NK cell subsets were defined based on expression levels of CD56 and CD16, which varied in distribution across blood and tissue NK cells (representative distributions for each tissue shown in **Figure 1B**).

NK cells made up 2-7% of lymphocytes in peripheral PB, BM, spleen, liver, jejunum, and lung tissues and less than 1% of lymphocytes in LNs (**Figure 1C**). PB, BM, and lung contained predominantly CD16+ NK cells with fewer DN NK cells and very few CD56+ NK cells (**Figure 1D**). Spleen and liver showed nearly equal proportions of DN and CD16+ NK cells with very few CD56+ NK cells. In contrast, LN samples showed primarily DN and CD56+ NK cells with very few CD16+ NK cells. In a single jejunum sample, the majority of NK cells were DN, with some CD16+ and very few CD56+ NK cells. These results align with recently reported data showing tissue-specific distribution and phenotypic characteristics of NK cells in humans (8) and non-human primates (14).

### CD16+ and DN NK Cells Have Longer Retention Time in Blood Than CD56+ NK Cells

**Figure 2:**
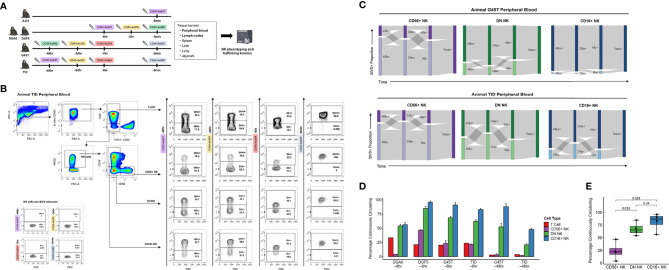
NK cell dynamics in peripheral blood. **(A)** Experimental time course of SIVS injections for five animals. Syringe icons indicate SIVS injections given at the time listed before euthanasia and blood sampling. Colored boxes indicate the fluorescently labeled CD45 antibody used. **(B)** Representative flow plots for animal TID which received the 48 hour SIVS time course. Staining for each of the four SIVS antibodies is shown for T cells, CD56+ NK cells, DN NK cells, and CD16+ NK cells as well as negative control NK cells sampled prior to SIVS infusion (bottom left). **(C)** Sankey diagram for animal G45T (top) and animal TID (bottom) which received the 48 hour SIVS time course. Cells were recovered from blood at time zero and the times listed are hours or minutes before time zero corresponding to the SIVS infusions shown in Panel **(A)** Bars show the proportion stained positive/negative for the SIVS antibodies administered at each timepoint and the gray chords show the fraction of cells that transition between each combination of positive and negative between time points. **(D)** Percentage of “Continuously Circulating” in each peripheral blood cell subset, defined as cells staining positive for all timepoint antibodies administered from the initial timepoint listed below the animal name through euthanasia and blood sampling. For DGA0 and DGF5, this is the fraction of cells stained positive for the SIVS antibodies given at -6hr, -2hr, and -5min. For “G45T -6hr” and “TID -6hr”, this is the fraction of cells stained positive for the -6hr and -5min SIVS antibodies. For “G45T -48hr” and “TID -48hr”, this is the fraction of cells stained positive for the -48hr, -24hr, -6hr and -5min SIVS antibodies. Bars show the mean and error bars show the standard error of the mean between 2 technical replicates for each cell sample. **(E)** The fraction of cells Continuously Circulating for up to 6 hours for each of the four animals in panel **(D)** Each dot shows the mean from one animal. The box shows interquartile range and the black line shows the median value for each NK cell subset. The whiskers extend to the furthest data point, and p-values shown were computed using a Wilcoxon test between each NK subset.

We administered anti-CD45 SIVS antibody infusions to five healthy RMs (**Figure 2A**). Animal ZJ31 received a single infusion 5 minutes before time zero when final blood sampling and tissue harvesting was performed. Animals DGA0 and DGF5 received SIVS antibodies 6 hours, 2 hours, and 5 minutes before time zero, and animal G45T and TID received a longer time course of SIVS antibodies 48 hours, 24 hours, 6 hours, and 5 minutes before time zero.

We stained PBMCs for different NK cell surface markers and quantified the SIVS antibody staining within CD56+, DN, and CD16+ NK cells and T cells (**Figure 2B**). We first validated that there is no background signal for the SIVS antibodies prior to SIVS infusions (**Figure 2B**, lower left). After defining positive and negative gates for each SIVS marker in each cell population, we analyzed the data for each animal’s NK cell populations individually using Sankey diagrams, which shows the percentage of cells positive and negative for each individual timed SIVS infusion over subsequent time points. For animals G45T and TID, receiving SIVS infusions over 48 hours, the majority of CD56+ NK cells recovered from PB at final collection were negative for the -6 hours infusion, indicating that they had recently emigrated from tissue to PB within the past 6 hours (**Figure 2C**). There was substantial trafficking of CD56+ NK cells between the vasculature and tissue compartments indicated by the fraction of cells changing from positive to negative or vice versa between subsequent SIVS infusions (gray flow lines). For DN NK cells, more cells were positive for the -6 hours antibody than for CD56+ NK cells, indicating PB localization at that time period, although approximately 30% of DN NK cells were recent emigrants from tissue within the last 6 hours before necropsy. There was some trafficking between tissue and vasculature for DN cells, though a considerable fraction (25-50%) of DN NK cells were retained in the vasculature for the full time course of SIVS infusions. For CD16+ NK cells, the majority of cells were positive for all SIVS infusions, indicating sustained residence in the vasculature for the 48 hour time course. For the animals DGA0 and DGF5 receiving shorter SIVS time courses, similar patterns in trafficking kinetics between NK subsets were found, with CD16+ NK cells having the longest retention time in blood, followed by DN NK cells, and CD56+ NK cells showing the most trafficking between blood and tissue (**Figure S1A**). These data, consistent across four animals, indicate that distinct NK cell subsets have differences in trafficking rates between PB and tissue at steady state.

We compared the percentage of cells which were “Continuously Circulating” (CC) i.e., retained in the vasculature, for each NK cell subset for each animal and for T cells. When considering timepoints going back to 6 hours before necropsy, all four animals had the same pattern with CD56+ NK cells having the lowest proportion of CC cells, CD16+ NK cells having the highest proportion, and DN NK cells intermediate between the two (**Figure 2D**). When considering all time points through -48 hours for animals G45T and TID, the same pattern held, indicating sustained residence in blood for the full time course of experiment i.e., 48 hours. We compared the fraction of CC cells between NK subsets using a Wilcoxon test with the four animals as biological replicates and found that DN and CD16+ NK cells had a higher percentage of continuously circulating cells than CD56+ NK cells (**Figure 2E**). Taken together, these results indicate that CD16+ and DN NK cells, which make up on average >95% of PB NK cells in RM (**Figure 1D**), are circulating continuously with an average residence time in the vasculature greater than six hours. Whereas the minor population of CD56+ NK cells in blood have a significantly shorter residence time and more commonly traffic between blood and tissue.

### NK Subsets Show Differential Trafficking Kinetics Into Lymph Nodes and Other Tissues

**Figure 3:**
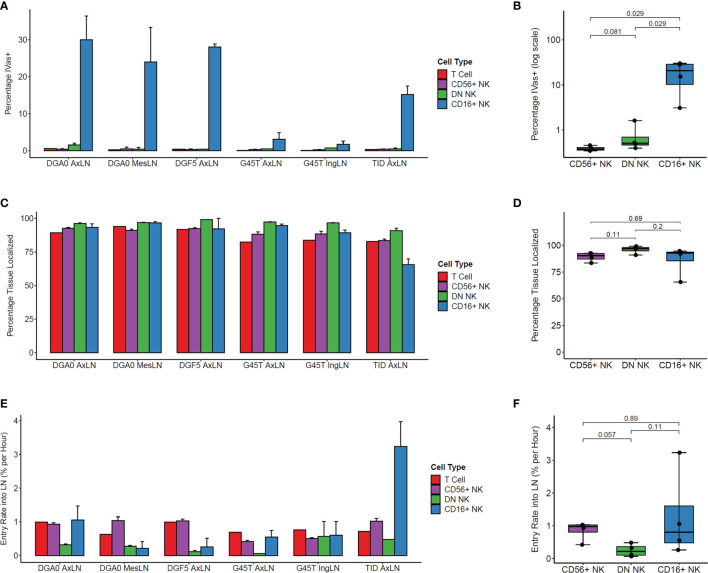
Kinetics of NK cell trafficking into lymph nodes. **(A)** The percentage IVas+ cells in each cell subset from LN samples harvested from the four animals at time zero. IVas+/- refers to the SIVS antibody given 5 minutes prior to tissue harvest. **(B)** The mean percentage IVas+ cells for each NK cell subset within the AxLN sample for each animal was compared (n = 4 for each subset). For all boxplots, each dot shows the mean from one animal’s AxLN sample. The box shows interquartile range and the black line shows the median value for each NK cell subset. The whiskers extend to the furthest data point, and p-values shown were computed using a Wilcoxon test between each NK subset. **(C)** The percentage of IVas- cells for each cell subset which are “Tissue Localized” (negative for all SIVS antibodies given up to 48hr-G45T and TID or 6hrs-DGF5 and DGA0 before tissue harvest). **(D)** The Tissue Localized fraction for each AxLN sample in Panel **(C)** was compared using a Wilcoxon test (n = 4 for each subset). **(E)** The percentage of IVas- cells which are positive for the SIVS 6hr antibody for each subset divided by 6 hours to give an entry rate into the lymph node for each sample. **(F)** The mean entry rate for each AxLN sample is taken from panel **(E)** and compared between subsets using a Wilcoxon test (n = 4 for each subset). AxLN, Axillary Lymph Node; MesLN, Mesenteric Lymph Node; IngLN, Inguinal Lymph Node.

We determined the positional history of lymphocytes isolated from LN samples at tissue harvest. Across multiple LN samples from three animals, CD16+ NK cells showed the highest percentage of IVas+ stained by the SIVS infusion given 5 minutes before LN biopsy (**Figure 3A**). This difference between CD16+ and both CD56+ and DN NK cells was statistically significant in axillary lymph node (AxLN) samples from the four animals analyzed (**Figure 3B**). Though IVas+ NK cells in the LN could represent blood contamination, the NK cell phenotype of the LN IVas+ compartment more closely resembled the LN IVas- compartment than the blood compartment in most animals (**Figures S2B–D**). IVas+ cells in the LN have been shown to be closer to blood vessels (38) so our results indicate that CD16+ NK cells, which make up on average less than 10% of cells in LN (**Figure 1D**), may be preferentially localized to the cortex of the LN closer to the blood vessels. For assessing tissue localization and cell entry rates into LN, we considered only IVas- cells.

The IVas- NK cells for all three subsets showed high percentage of Tissue Localized (TL) cells, defined as negative for all SIVS antibody infusions (**Figure 3C**). For all LN samples tested, DN NK cells had the highest fraction of TL cells, though the difference was not statistically significant when comparing the AxLN samples from the four animals (**Figure 3D**). We next assessed the entry rate into LN, defined as the percentage of IVas- cells which were positive for the -6 hour infusion divided by six hours to give an entry rate in percentage of cells per hour. For the AxLN samples from all four animals, DN NK cells had the slowest rate of entry into the LN (**Figure 3E**). However, this difference was not statistically significant with our sample size of four AxLN samples (**Figure 3F**). The high variation in LN entry rate of CD16+ NK cells is likely due to the lower cell number, considering it is a minor population in LN and for some animals, a large percentage of CD16+ NK cells are IVas+ so were excluded from entry rate calculations. Taken together, these results indicate that for the two major NK cell populations in the LN, CD56+ and DN NK cells, the majority of cells are localized to the LN with a residence time greater than six hours, and that the trafficking rate from blood into the LN is less than 1% of LN cells per hour.

We repeated the same analysis on spleen samples from animals DGF5, G45T, and ZJ31 (**Figure S3**). We found high percentages of IVas+ cells across all three NK cell subsets and T cells (**Figure S3A**) which was expected given that spleen capillaries harbor discontinuous endothelial cells and as such are termed sinusoidal capillaries (44). Among the IVas- cells in the spleen, CD16+ NK cells had the lowest fraction of TL cells (**Figure S3B**) and the highest entry rate into the spleen (**Figure S3C**) out of the three NK subsets. Comparison of the NK phenotype between PB, spleen IVas+ cells, and spleen IVas- cells supported the notion that the IVas+ cells may occupy a distinct perivascular niche within the spleen, since they did not match the phenotype of PB NK cells (**Figures S3D, E**).

We analyzed NK cell trafficking kinetics into liver, lung, and jejunum, though we had fewer biological replicates for these tissues. In our liver sample, most NK cells were IVas+, which was expected given that the liver contains primarily sinusoidal capillaries. The liver IVas+ cells had a distinct NK phenotype from blood cells in the same animal (**Figure S4A**), indicating that a large fraction of NK cells in liver may occupy a perivascular niche, as previously reported for T cells in the liver (38). Within our lung sample, the majority of DN and CD16 NK cells were IVas+, while the majority of CD56 NK cells were IVas-, indicating a possible difference in localization within lung tissue (**Figure S4B**). The majority of IVas- CD16+ NK cells were Recent Immigrants (RI) to the lung (defined as IVas- but SIVS+ for one of the previous time points), while the majority of IVas- CD56 and DN NK cells were TL. Again, the IVas+ lung cells differed in NK phenotype from the PB sample from the same animal, suggesting the presence of a perivascular niche. Within our jejunum sample, there were very few CD56+ NK cells, and almost all NK cells were IVas- (**Figure S4C**). Of the IVas- NK cells, the majority of DN NK cells were TL within the jejunum while the majority of CD16+ NK cells were RI. Our results suggest that different NK cell subsets may exhibit different localization patterns and residence times within lymphoid and non-lymphoid tissues.

### Surface Levels of CD69 Are Associated With NK Cell Retention in the Lymph Node

**Figure 4:**
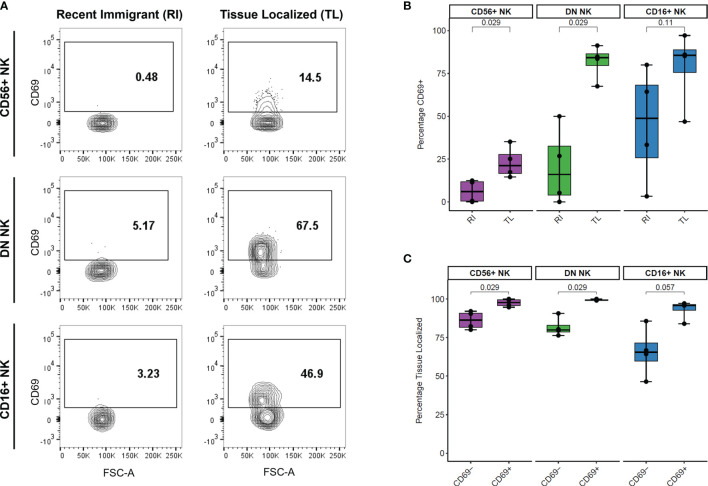
Association between CD69 surface levels and tissue localization in LN NK cells. **(A)** Representative CD69 surface marker levels compared between Recent Immigrant (RI) and Tissue Localized (TL) cells within each NK subset from animal TID axillary lymph node sample. RI cells are IVAS- but stained positive for at least one other timepoint, while TL cells stained negative for all SIVS infusions. **(B)** Comparison of the percentage of cells positive for CD69 between RI and TL NK cells from each NK subset from four animals axillary lymph node. Each dot shows the percentage in one animal’s AxLN sample (n = 4 for each NK subset). The box shows interquartile ranges, and the whiskers extend to the furthest data point. P-values are from a Wilcoxon test comparing each NK subset. **(C)** Comparison of the proportion of cells which were Tissue Localized (TL) within the CD69- and CD69+ subset from each NK cell subset from four animals axillary lymph node sample. Box plots are the same as in panel **(B)** and p-values are from a Wilcoxon test comparing each NK subset (n = 4 for each subset).

We analyzed the surface levels of six markers associated with NK cell trafficking and/or activation (CD49a, CD161, NKp46, CCR7, CD69, and CXCR3) as well as the intracellular proliferation marker KI-67 across LN samples. We compared the percentage of cells positive for each marker between recent immigrant (RI) and tissue-localized (TL) cells from each NK cell subset and found that the percentage of CD69+ cells was higher in TL than RI NK cells (**Figure 4A**). This difference was statistically significant for the two major populations of LN NK cells, CD56+ and DN NK cells, (n=4 AxLN samples) (**Figure 4B**). Additionally, we quantified the percentage of LN NK cells which were TL within the CD69- and CD69+ fraction of each NK cell subset (**Figure 4C**). Our results indicate that CD69 is a good marker for NK cell retention in the LN, with >99% of CD69+ DN NK cells, 94-100% of CD69+ CD56+ NK cells, and 84-97% of CD69+ CD16+ NK cells showing sustained residence in the LN for the full extent of our experiment (at least 6 hours, and up to 48 hours). Taken together, these results support the use of CD69 as a tissue residence marker for NK cells in the LN. The other five surface markers and KI-67 did not show any statistically significant difference between RI and TL NK cells in the LN (**Figure S5**).

### CD16+ and DN NK Cells Are Retained in the Vasculature During Regeneration of the Mature NK Cell Compartment After CD16 Depletion

**Figure 5:**
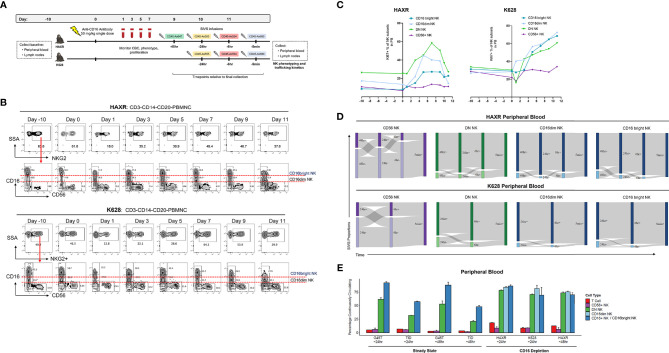
CD16 depletion. **(A)** Experimental diagram of tissue sampling, administration of CD16-depleting antibody, and time course of SIVS infusions. **(B)** Percentage of NKG2+ cells (top) and CD56/CD16 distribution (bottom) after CD16 depletion for the two animals. Definition of CD16^dim^ and CD16^bright^ NK subsets are shown. Day 0 samples were taken immediately prior to anti-CD16 antibody administration. **(C)** Percentage KI-67+ proliferating NK cells for each subset over the time course of CD16 depletion for the two animals. **(D)** Sankey diagram showing the proportion of PB NK cells stained positive/negative at each timepoint for CD16 depletion animals HAXR and K628. The times listed are hours or minutes before the final collection. **(E)** “Continuously circulating” is defined as staining positive for all timepoints up to the timepoint listed below the animal name. Bars show the mean and error bars show the standard error of the mean between 2 technical replicates for each NK cell sample. For CD16 depletion animals, the CD16+ NK cells are broken up into CD16^dim^ and CD16^bright^ NK as shown in panel **(B)**.

To investigate NK cell dynamics during NK regeneration, we transiently depleted mature CD16+ NK cells in two animals and then performed a SIVS experiment at the peak of NK regeneration as assessed by maximal cell cycling in the PB. As previously shown, CD16 depletion can efficiently deplete CD16+ NK cell cells (35, 40), and clonal patterns suggest that CD16+ NK cell regenerate from a proliferating CD16^dim^ subset (35). After collecting baseline PB and LN samples, we administered a single dose of anti-CD16 depleting antibody (**Figure 5A**). We collected PB daily and monitored the NK cell numbers and proliferation rates to select a time point with high proliferation to start the SIVS infusions. Following CD16+ NK depletion, the proportion of NKG2+ cells decreased sharply and then began to rebound after three days (**Figure 5B**). The surface levels of CD16 decreased, and we defined both CD16^dim^ and CD16^bright^ populations of CD16+ NK cells. The DN NK cells increased in proliferation at day three after antibody administration, followed by an increase in the proliferation of the CD16^dim^ and CD16^bright^ NK cells respectively (**Figure 5C**). There was no increase in proliferation within the CD56+ NK cell subset in blood in animal HAXR and a very mild increase in animal K628 (**Figure 5C**). We checked the levels of the NK cell degranulation marker CD107a and early activation marker CD69 surface levels throughout the CD16 depletion (**Figure S6A**). We found very low numbers of CD107a+ NK cells throughout the time course and a transient increase in CD69+ percentage after antibody administrations, which returned to approximately baseline levels by day nine.

We began SIVS antibody infusion nine days after anti-CD16 administration, based on a high proliferation rate at that time point of DN and CD16^dim^ NK cells, which represent putative precursors to CD16+ NK cells (35). We administered SIVS infusions at 48 hours, 24 hours, 6 hours, and 5 minutes before PB and tissue collection in animal HAXR (**Figure 5A**). We attempted to follow the same SIVS time course for animal K628, but due to extravascular extravasation of the -48 hour SIVS infusion, only the -24 hour, -6 hour, and -5 minute SIVS infusions were interpretable. During recovery from CD16 depletion, CD56+ NK cells showed a similar dynamic in terms of trafficking between blood and tissue (**Figure 5D**) as compared to steady state (**Figure 2C**). Despite the fact that CD16^bright^ NK cells were being actively regenerated at the time of SIVS infusions, the retention time of DN, CD16^dim^, and CD16^bright^ NK cells in the blood was very high, with all three subsets having high SIVS+ percentage at each timepoint back to 48 hours (HAXR) or 24hr (K628) before tissue collection. The fraction of cells that were continuously circulating was low for CD56+ cells and high for CD16+ (CD16^dim^ and CD16^bright^), similar to what was observed in steady state animals with the longer SIVS time course (**Figure 5E**). DN NK cells showed a higher percentage of continuously circulating cells in CD16 depletion animals as compared to steady state animals. This indicates that the regeneration of mature CD16^bright^ NK cells may be taking place peripherally within blood or from cells in the perivascular space of tissue, which may also be tagged by the infused antibody, not recruited from tissue resident NK cell populations.

Within the LN, the CD16 depletion animal shared the same pattern of IVas+ frequencies as the steady state animals, with CD16^dim^ and CD16^bright^ NK cells having the highest IVas+ frequency. The IVas+ percentage in these two subsets was higher than in CD16+ NK cells in steady state animals (**Figure S6B**). Of the IVas- cells in LN, there was a decrease in the proportion of tissue localized CD56+ NK cells and an increase in the LN entry rate of multiple NK subsets as compared to steady state animals (**Figures S6C,D**). This indicates that NK cells may be trafficking to and from the LN more dynamically during regeneration of the CD16 NK cell compartment.

## Discussion

We have utilized a powerful intravascular staining approach to study *in vivo* trafficking kinetics of NK cell subsets for the first time, focusing on a non-human primate model relevant to human biology. We found that CD16+ NK cells had significantly longer residence times in peripheral blood than CD56+ NK cells, and DN NK cells had an intermediate residence time. This pattern was conserved across four steady state animals and two animals which received CD16 depleting antibody. It has been previously observed that CD16+ NK cells express lower levels of key homing molecules CCR7 and CD62L than CD56+ NK cells (6, 7). These molecules bind to ligands on high endothelial venules permitting travel into LNs. To our knowledge, our findings provide the first *in vivo* quantitative demonstration that CD16+ NK cells are not transiting through secondary lymphoid organs as frequently as CD56+ NK cells at steady state. Our data also imply that, in steady state conditions, most blood CD16+ NK cells are not frequently trafficking in and out of other tissues or undiscovered reservoirs, at least those where they would be sequestered from blood circulation. Subset-specific localization and trafficking kinetics have been recently demonstrated in T cells, with the finding that a subset of highly cytotoxic human effector memory CD8+ T cells were selectively localized within the vasculature and remained there for months in multiple sclerosis (MS) patients who received a drug which inhibits sphingosine-1-phosphate (S1P) dependent tissue egress (45). NK cells are also dependent on the S1P receptor for homing during steady state and inflammation (46), and in MS patients receiving S1P blockade, the number and proportion of CD56^bright^ but not CD56^dim^ NK cells in PB were reduced (47, 48). Our data indicate a more dynamic redistribution of CD56+ NK cells between blood and tissue as compared to CD16+ NK cells, which helps to explain this clinical phenomenon. The selective retention of CD16+ NK cells, which are the more cytotoxic NK subset, and cytotoxic CD8+ effector memory T cells in the vasculature may be part of a conserved mechanism to protect tissues from damage under normal homeostasis (45).

We also found that NK subsets had different localization and trafficking patterns within LN and other lymphoid and non-lymphoid tissues. Very few CD56+ and DN NK cells in the LN were stained by the infusion given 5 minutes before tissue collection, whereas 5-30% of CD16+ NK cells were IVas+ in steady state animals. This indicates that CD16+ NK cells may occupy a distinct perivascular niche within the lymph node and are likely found in the cortex and paracortex close to CD31+ blood vessels (38). At steady state, NK cells have been found in the parafollicular regions of the T cell zone in human (49, 50) and mouse (51) LN but the localization of specific NK subsets within LN has been poorly studied, possibly due to the low number of CD16+ NK cells in LN. The two major populations of NK cells in the LN, CD56+ and DN, exhibited a high percentage of tissue localization over the time course of our experiment and a LN entry rate of 0-1% of LN cells per hour. We also found differences in the trafficking pattern of NK cell subsets in spleen as well as non-lymphoid organs (liver, lung, and jejunum). In liver, lung, and jejunum, the IVas+ NK phenotype was distinct from that of blood further supporting the notion of a distinct perivascular compartment in these tissues (38, 52).

Combining SIVS technology with analysis of NK cell surface markers revealed a high percentage of CD69+ cells within NK cells localized to the LN. CD69 is a transmembrane receptor of the calcium dependent lectin superfamily. In addition to being an early activation marker for NK cells, CD69 has a known role in promoting retention of lymphocytes in lymphoid tissues through suppression of sphingosine-1-phospate receptor 1 (53–55) and has been described as a tissue residence marker for T cells (56, 57). We found that the CD69+ subset of LN NK cells was significantly enriched for cells which had been retained in the LN for our full SIVS experiments (up to 48 hours). This supports the use of CD69 as a marker for LN-resident NK cells in rhesus macaque and provides evidence that CD69+ NK cells are not only found at high proportion in tissues but are preferentially retained within the lymph node.

We depleted mature CD16+ NK cells from two animals and performed SIVS infusions during NK cell recovery to shed light on possible developmental relationships and localization of regeneration of NK cell subsets. We hypothesized that if CD16+ NK cells were being formed from precursor cells in secondary lymphoid tissues, then a high proportion of newly formed CD16+ NK cells during recovery would be negative for SIVS infusions given several hours before collection. However, we found that DN NK cells and both subsets of CD16+ NK cells (CD16^dim^ and CD16^bright^) had a large proportion of cells continuously circulating in blood during recovery, at similar levels to steady state animals. This suggests that within the span of our SIVS experiment (48 hours), regeneration of CD16+ NK cells was either occurring from precursor cells within the blood or cells within the perivascular region of a tissue, such as the spleen or liver, which would also be tagged by the infused antibodies. We did not observe any peak in proliferation of CD56+ NK subset in blood during CD16 regeneration, and only observed an increase in proliferation of CD56+ NK cells in LN in one animal (**Figure S6E**), which calls into question a direct continuous differentiation from CD56+ to CD16+ at least in the context of recovery from mature CD16 NK cell depletion, going along with prior observations regarding NK cell life histories based on genetic barcoding studies (35).

There are limitations to our study. First, we could not obtain large sample numbers for tissues that require sacrifice or invasive biopsy of non-human primates such as liver, lung, and jejunum, so we have focused our analysis on blood and LN, which can be easily sampled from live animals. Second, we observed a reduction in NK cell frequency (especially in the CD16+ subset) after administration of SIVS antibodies to animals following CD16 depletion, likely reflecting an immune response to the mouse-derived IgG portion of the antibody. Although this sensitization may lead to increased NK cell death, it does not limit our ability to interpret the positional history of the cells which we recovered at the conclusion of the experiment.

Recent research has provided insights into distinct functions and patterns of tissue localization of human NK cell subsets (8–14). Here, we have added *in vivo* demonstration of differential rates of retention in blood and trafficking into tissues between NK cell subsets in a non-human primate model relevant to human biology. There is much interest in tracking NK cells in the context of adoptive transfer therapies (58–62). Our data suggest that differences in tissue localization and trafficking patterns between subsets of NK cells should be considered to understand the potential therapeutic applications of these promising cell therapies.

## Data Availability Statement

The datasets presented in this study can be found in online repositories. The names of the repository/repositories and accession number(s) can be found below: https://github.com/dunbarlabNIH/SIVS_NK.

## Ethics Statement

The animal study was reviewed and approved by National Institute of Allergy and Infectious Disease and National Heart Lung and Blood Institute Animal Care and Use Committees.

## Author Contributions

Conceptualization, RM, CW, EP, MR, and CD. Methodology, EP, CW, RM, and DSJA. Investigation, EP, CW, RM, and DMA. Formal analysis, RM, CW, and EP. Visualization, RM and CW. Supervision, EP, CW, SH, MR, and CD. Writing – original draft, RM, CW, and CD. Writing – review and editing, RM, CW, EP, DMA, DSJA, SH, MR, and CD. All authors contributed to the article and approved the submitted version.

## Funding

All work was supported by the intramural programs of the National Heart, Lung and Blood Institute and the Vaccine Research Center of the National Allergy and Infectious Diseases Institute.

## Conflict of Interest

The authors declare that the research was conducted in the absence of any commercial or financial relationships that could be construed as a potential conflict of interest.

## Publisher’s Note

All claims expressed in this article are solely those of the authors and do not necessarily represent those of their affiliated organizations, or those of the publisher, the editors and the reviewers. Any product that may be evaluated in this article, or claim that may be made by its manufacturer, is not guaranteed or endorsed by the publisher.
