# Supplementary material for: Tissue Trafficking Kinetics of Rhesus Macaque Natural Killer Cells Measured by Serial Intravascular Staining
Source: Front Immunol. 2022 Jan 11;12:772332. doi: 10.3389/fimmu.2021.772332 (PMC8790741; doi:10.3389/fimmu.2021.772332)
Supplement: Supplementary file 1 [file DataSheet_1.pdf]

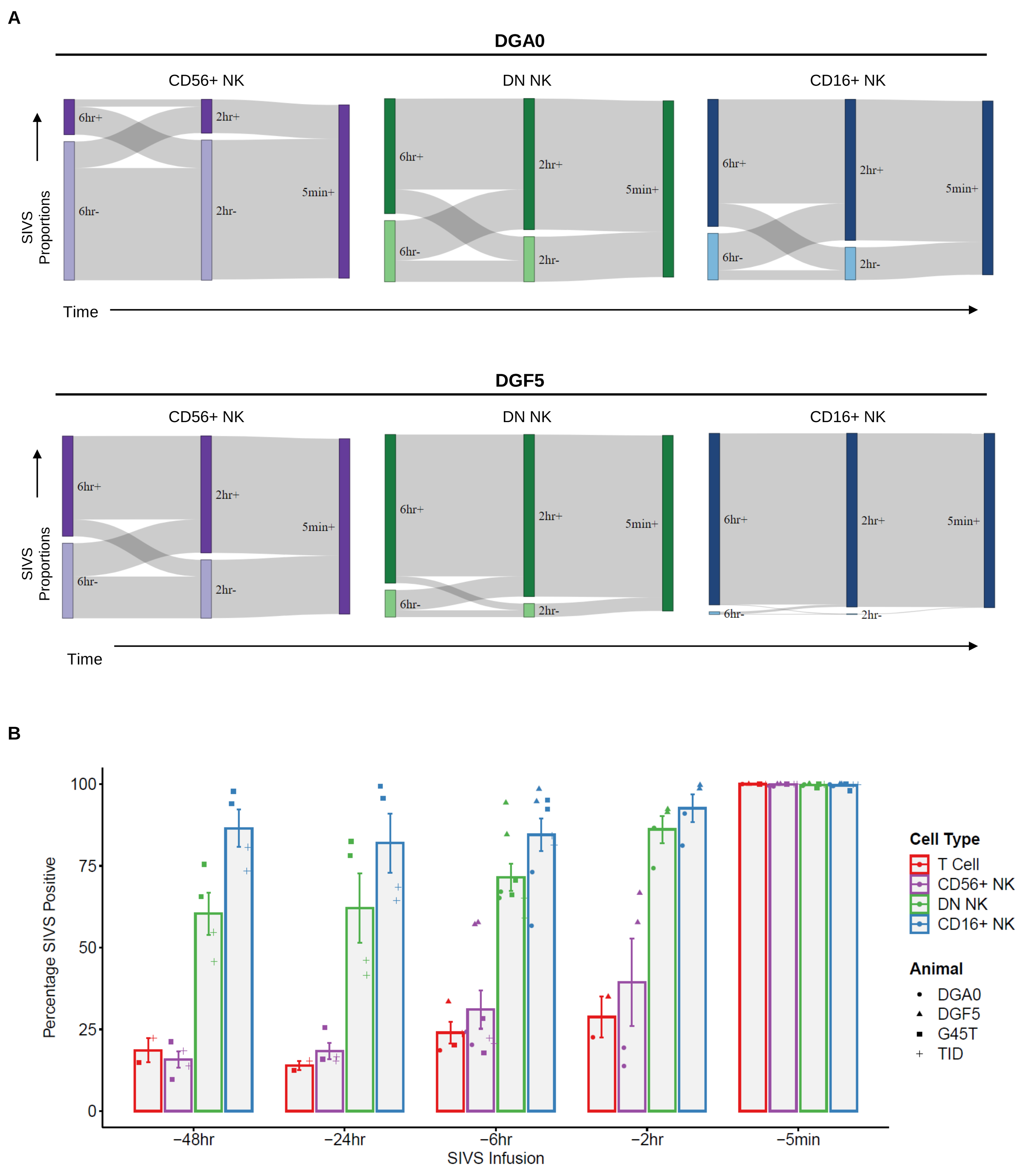

Supplementary Figure 1: NK Cell Dynamics in Peripheral Blood

A) Sankey diagram for animal DGA0 and DGF5. Bars show the proportion stained positive/negative for the SIVS antibody administered at each timepoint before tissue harvest and the gray chords show the fraction of cells that transition between each combination of positive and negative when moving to the next time point.

B) Percentage positive for each SIVS timepoint antibody for all SIVS animals. Two technical replicates are shown for each NK cell sample. Bar plot shows mean and error bars show standard error of the mean for each cell type and timepoint. The timepoints are listed as hours or minutes before tissue harvest.

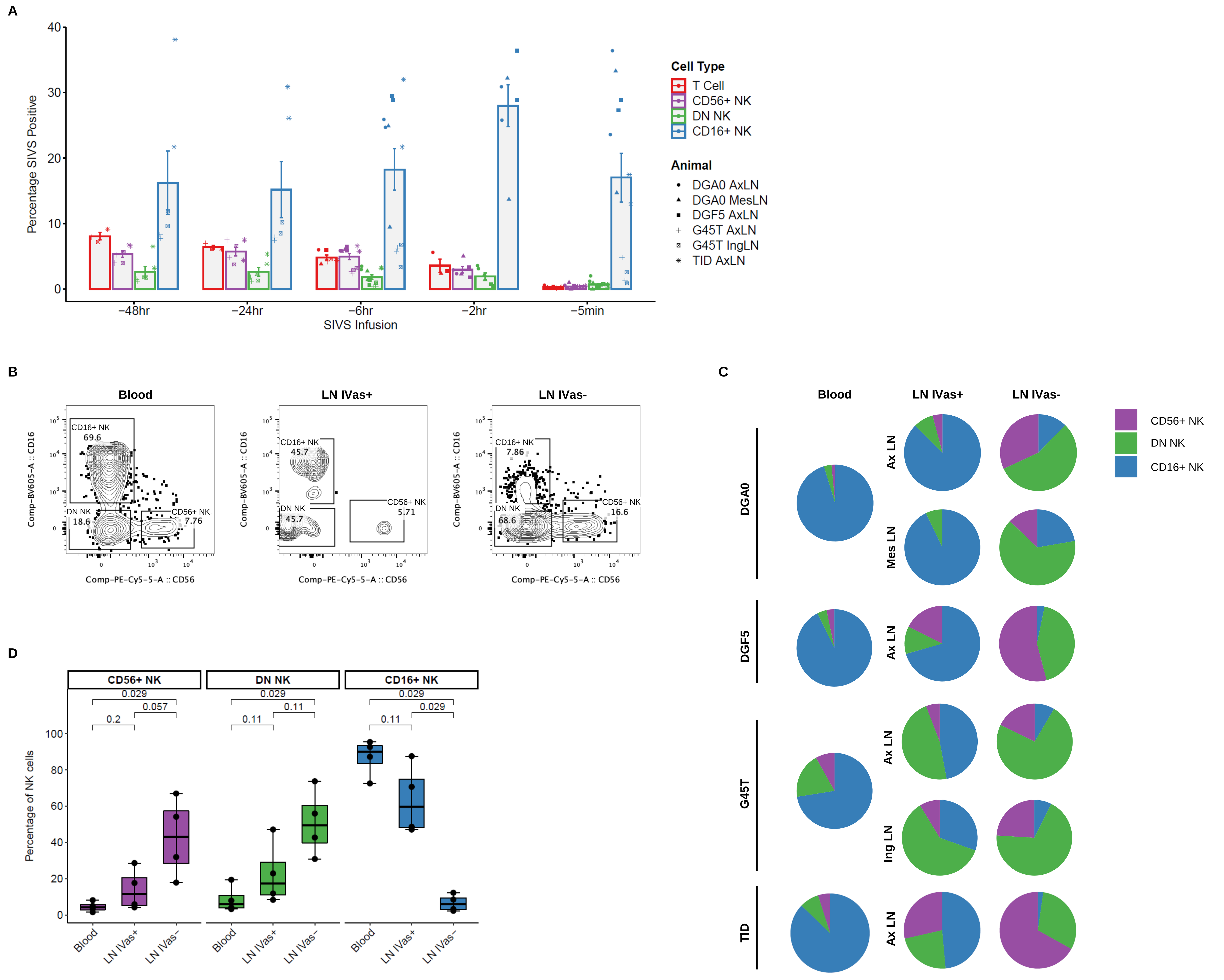

Supplemental Figure 2: Kinetics of NK Cell Trafficking into Lymph Nodes

(A) Percentage SIVS positive for SIVS antibodies given at each timepoint for all LN samples harvested at euthanasia. Two technical replicates are shown for each NK cell sample. Bar plot shows mean and error bars show standard error of the mean for each cell type and timepoint. The timepoints are listed as hours or minutes SIVS antibody was injected before tissue harvest.

(B) NK cell phenotype from animal G45T's blood, Axillary LN IVas+ (positive for SIVS antibody given 5 minutes before euthanasia), and Axillary LN IVas-.

(C) Proportion of NK cell subsets for blood and IVAS positive and negative compartments from lymph nodes samples of four animals. The blood phenotype is only shown once as it can be compared to both lymph node sample phenotypes.

(D) The percentage of each NK cell subset compared between each compartment with p-values shown from a Wilcoxon test (n = 4 for each comparison). The box shows interquartile ranges and the line shows the median value. Whiskers extend to the farthest data points.

AxLN, Axillary Lymph Node; MesLN, Mesenteric Lymph Node; IngLN, Inguinal Lymph Node

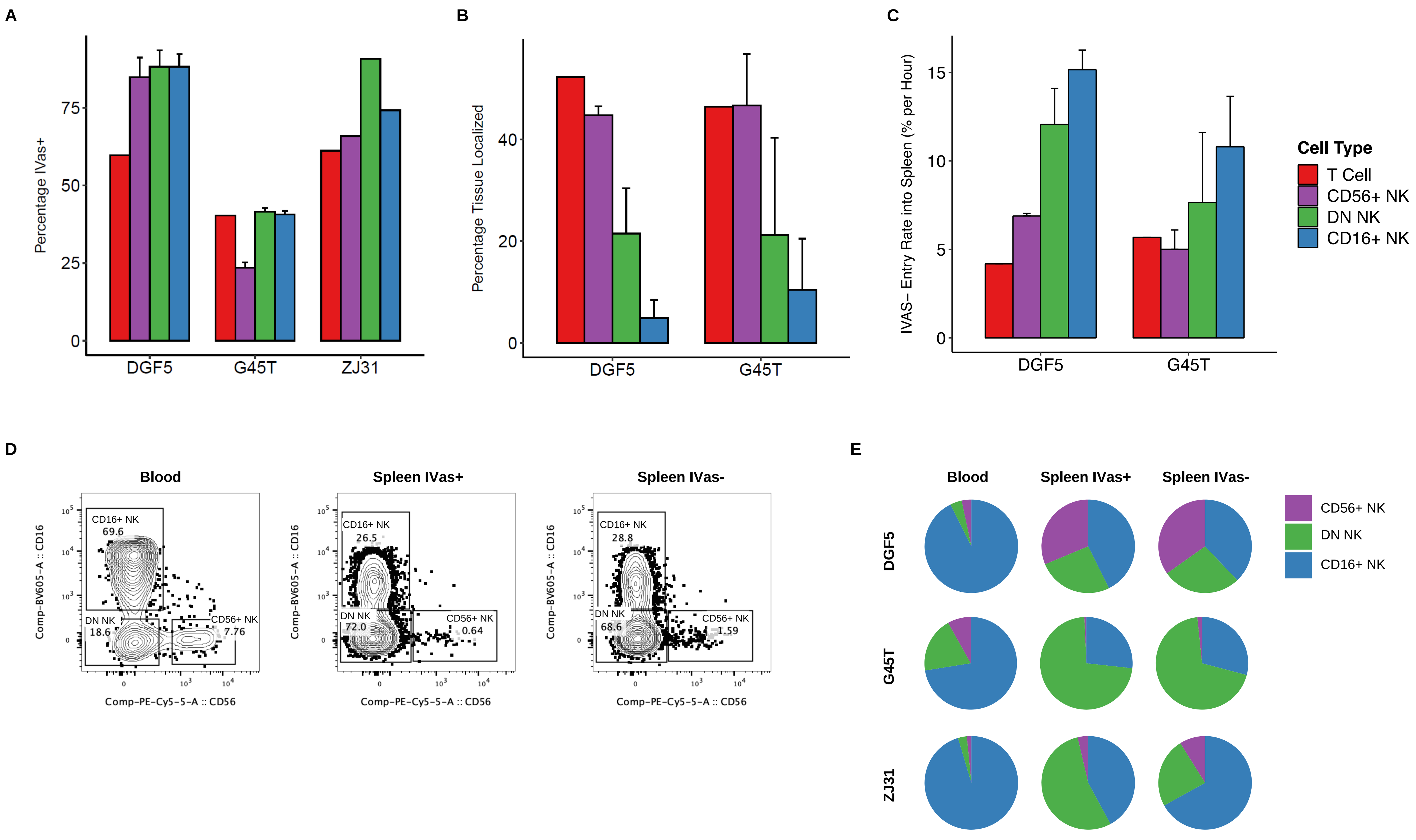

Supplemental Figure 3: Kinetics of NK Cell Trafficking into Spleen

(A) The percentage IVas+ of each cell subset from each spleen sample. IVas+/- refers to the SIVS antibody given at 5 minutes prior to tissue harvest. Bars show the mean and error bars show the standard error of the mean between 2 technical replicates for NK cell samples.

(B) The percentage of IVas- cells for each cell subset which are “Tissue Localized” (negative for all other SIVS antibodies). Bars show the mean and error bars show the standard error of the mean between 2 technical replicates for each NK cell sample.

(C) The percentage of IVas- cells which are positive for the 6hr SIVS antibody for each subset divided by 6 hours to give an entry rate into the lymph node for each sample. Bars show the mean and error bars show the standard error of the mean between 2 technical replicates for each NK cell sample.

(D) NK cell phenotype from animal G45T blood, spleen IVas+, and spleen IVas-.

(E) Proportion of NK cell subsets for blood and IVas positive and negative compartments from spleen samples of three animals.

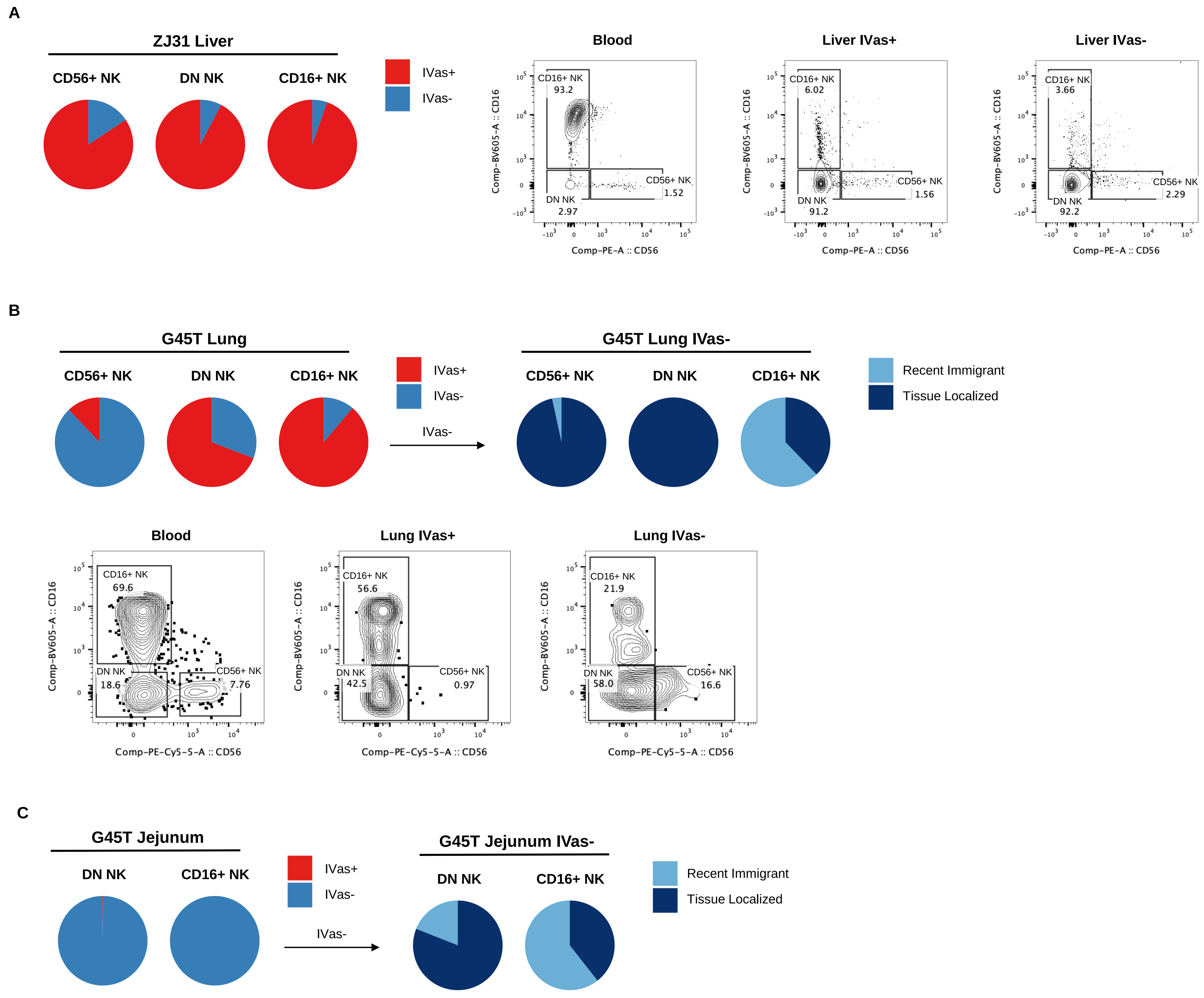

Supplemental Figure 4: Kinetics of NK Cell Trafficking into Liver, Lung, and Jejunum

A) Left: IVas+ percentage (positive for SIVS antibody given 5 minutes before tissue harvest) within each NK cell subset for the ZJ31 liver sample. Right: comparison of NK phenotype between blood, liver IVas+ cells, and liver IVas- cells for the animal ZJ31.

B) Top left: breakdown of IVas+ percentage within each NK cell subset for the G45T lung sample. Top right: from the IVas- fraction, the proportion of "Recent Immigrants", defined as negative for the 5min SIVS antibody but positive for any other SIVS antibody and "Tissue Localized", defined as negative for all SIVS antibodies. Bottom: comparison of NK phenotype between blood, lung IVas+ cells, and lung IVas- cells for the animal G45T.

C) Left: breakdown of IVas+ percentage within each NK cell subset for the G45T jejunum sample. Right: from the IVas- fraction, the proportion of "Recent Immigrants", defined as 5min- but positive for any other SIVS antibody and "Tissue Localized", defined as negative for all SIVS antibodies. The jejunum contained too few CD56+ NK cells to analyze.

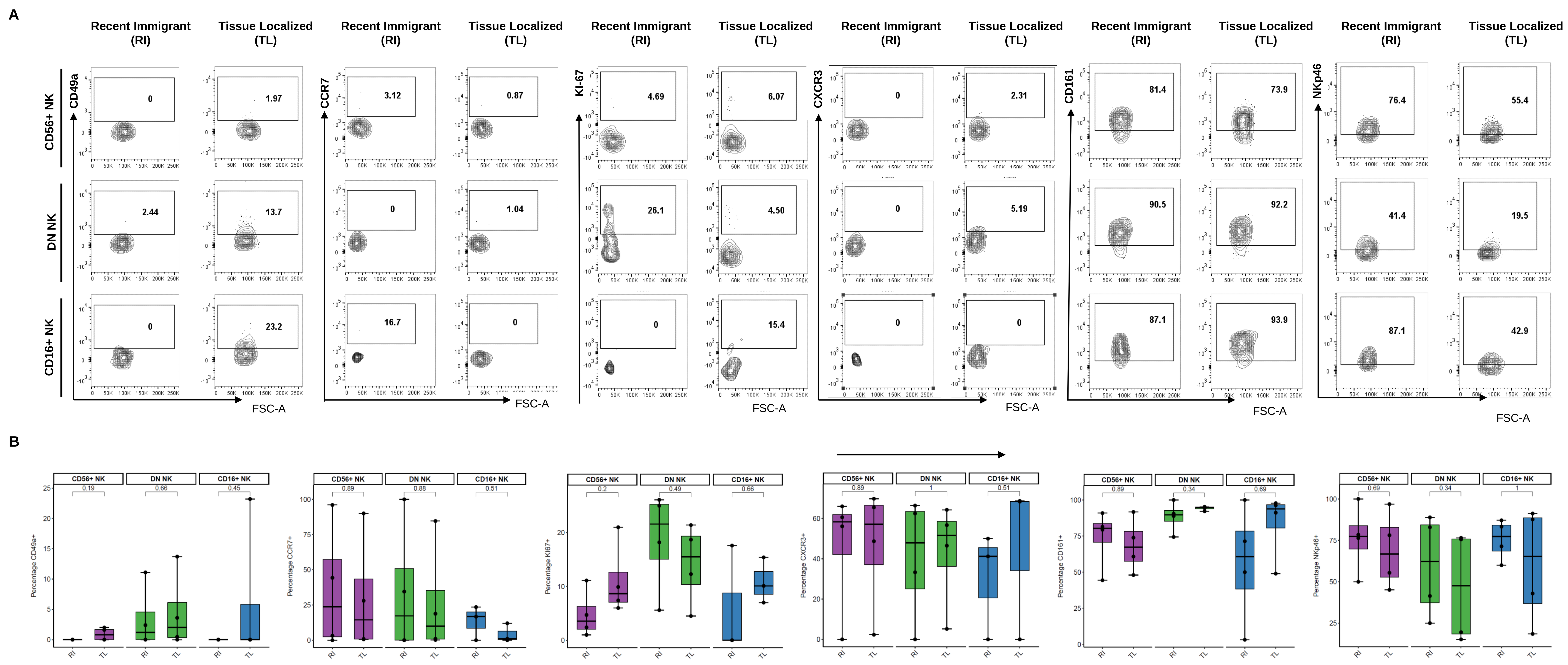

Supplemental Figure 5: Association between NK cell surface markers and tissue localization in LN NK cells

A) Representative flow plots in for CD49a, CCR7, KI-67, CXCR3, CD161, and NKp46 from animal TID axillary lymph node.

B) Summary of percentage positive of the corresponding surface maker from axillary lymph node samples from four animals. Boxes show the interquartile range, line shows the median value, and shislkers extend to the farthest data points. The p-values show the result of a Wilcoxon test between RI and TL cells for each subset (n = 4 for each comparison).

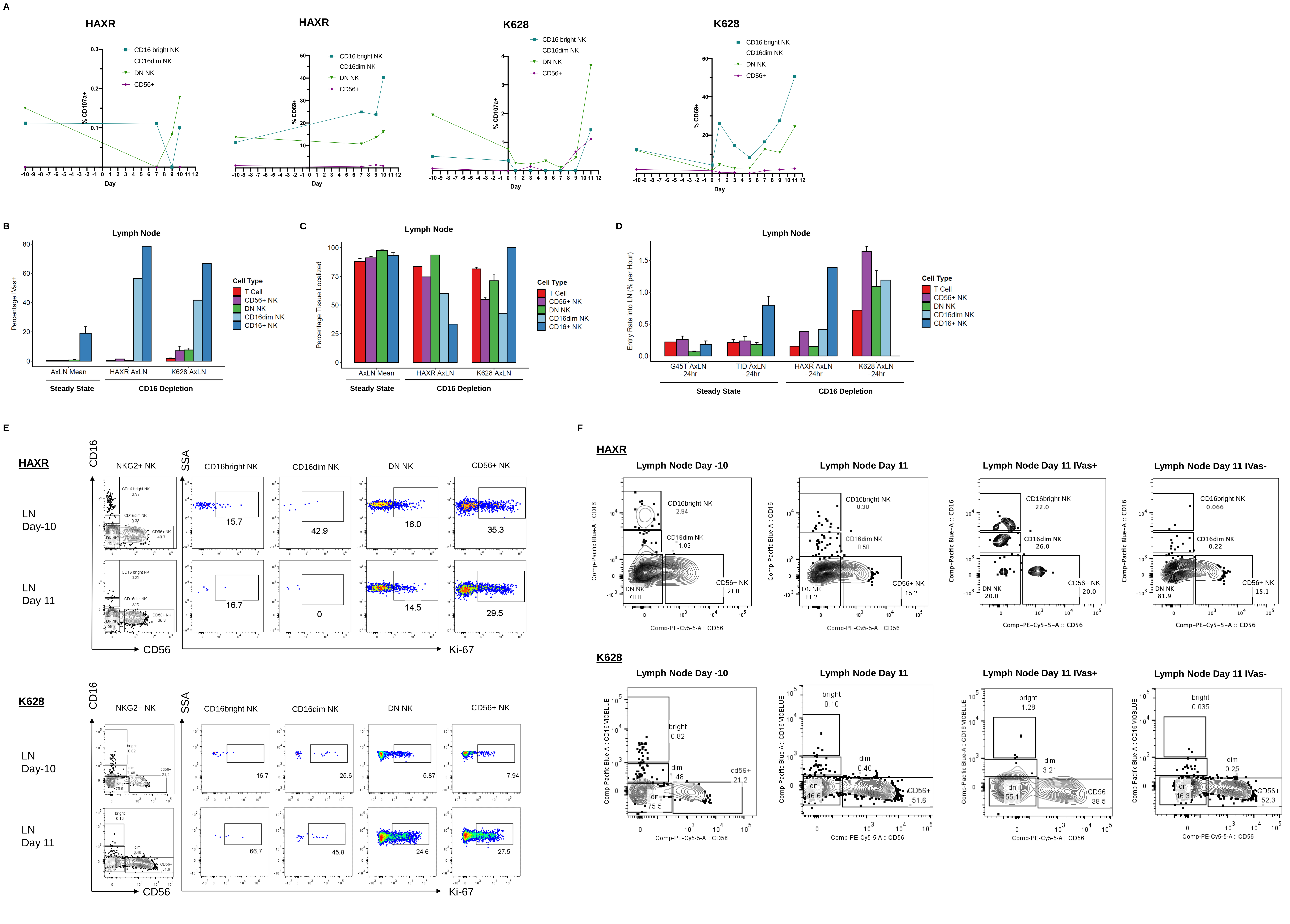

Supplementary Figure 6: CD16 Depletion

A) Percentage of cells positive for the NK degranulation marker CD107a and NK early activation marker CD69 for CD56+, DN, CD16dim, and CD16bright NK cells during recovery from CD16 depletion for animals HAXR and K628.

B) Comparison of the percentage IVas+ cells in AxLN samples between steady state animals (average from animals DGA0, DGF5, G45T, and TID) and CD16 depletion animals HAXR and K628. IVas+ refers to cells which stained positive for the infusion given 5 minutes prior to tissue harvesting. For panels B, C, and D, bars show the mean between technical replicates and error bars show the standard error of the mean. Oonly one technical replicate was performed for HAXR AxLN sample and for K628 CD16dim and CD16bright cells due to low cell number.

C) Comparison of the percentage Tissue Localized between steady state animals (average from animals DGA0, DGF5, G45T, and TID) and CD16 depletion animals HAXR and K628. Tissue Localized means negative for all SIVS infusions.

D) Comparison of the LN entry rate per hour between steady state animals (G45T and TID) and CD16 depletion animals (HAXR and K628). The LN entry rate per hour is calculated as the fraction of IVas- (5min-) that are positive for the -24 hour infusion divided by 24. The -24 hour infusion was used because it is the longest timepoint shared between all four animals.

E) KI-67+ fraction of each NK cell subset in AxLN Day -10 baseline and Day 11 (post-CD16 depletion and SIVS) samples from animals HAXR and K628.

F) Comparison of NK phenotype in lymph node from baseline versus final collection IVas+ and IVas- cells for CD16 depletion animal HAXR and K628.

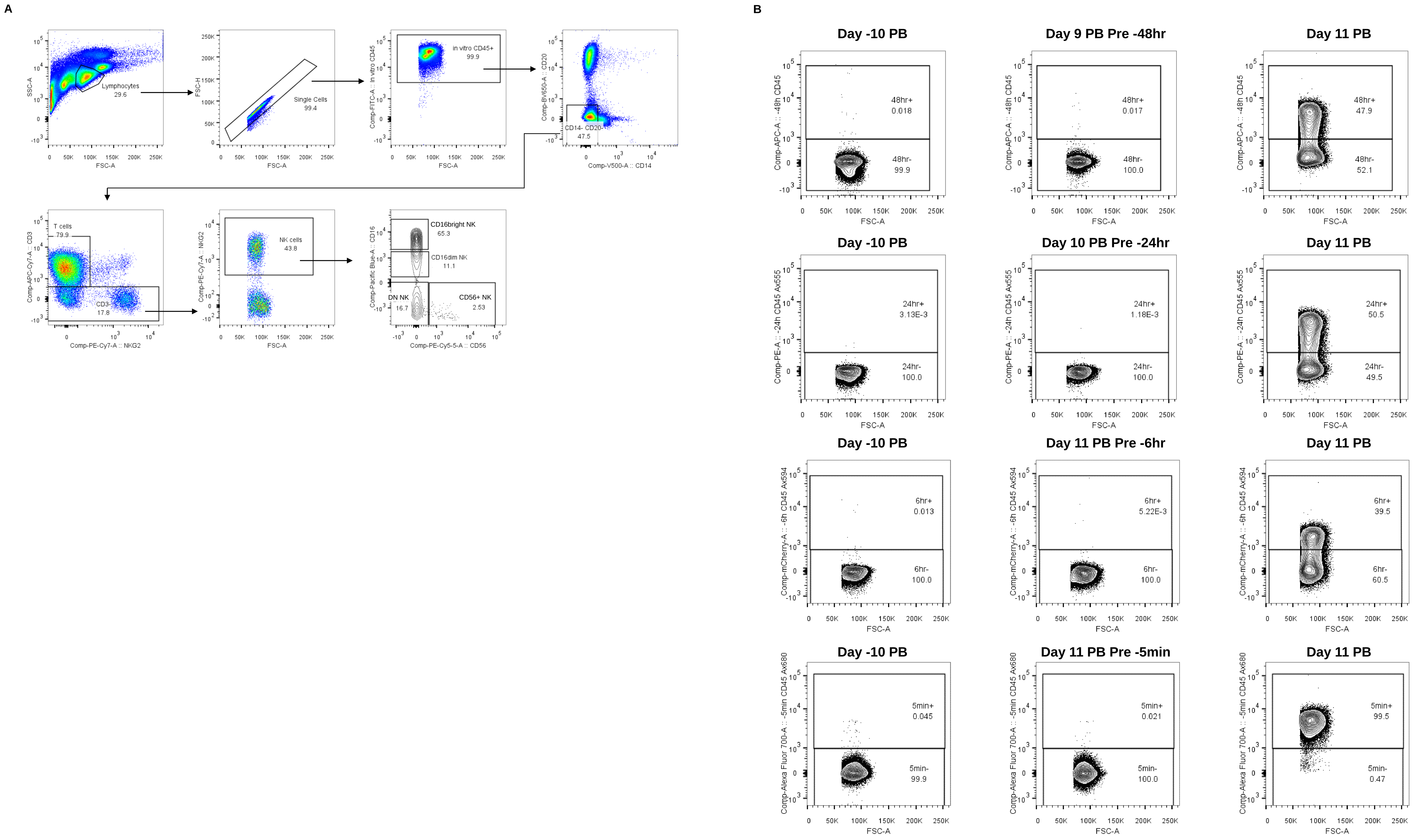

Supplementary Figure 7: Example gating scheme and negative controls from the CD16 depletion experiment

(A) Gating scheme for identification of T cells and NK cell subsets in CD16 depletion experiment. Example flow plots from animal HAXR.

(B) Staining for each SIVS antibody within lymphocytes from the Day -10 PB sample (negative control), the PB sample taken immediately before infusion (technical control), and the final collection sample for animal HAXR.

Table S1. Antibody Information

Panel #1

Fluorochrome	Marker	Clone	Company	Catalog Number	Volume (uL) per 0.5 - 2 million cells	Note
APC	CD45-Ax647	Custom made, see methods			0.2	Added *in vitro* for animals G45T and HAXR.
PE	CD45-Ax555	Custom made, see methods			0.2	
FITC	CD45-Ax488	Custom made, see methods			0.2	
mCherry	CD45-Ax594	Custom made, see methods			0.2	
Alexa 700	CD45-Alex680	Custom made, see methods			0.2	Added *in vitro* for animals DGA0 and DGF5.
APC-cy7	CD3	SP34-2	BD Pharmingen	557757	4	
APC-cy7	CD14	M5E2	BioLegend	301820	4	
APC-cy7	CD20	L27	BD	335794	4	
PE-cy7	NKG2A	Z199	Beckman Couter	B10246	2	
BV605	CD16	3G8	BD Horizon	563172	2	
PE-CY5.5	CD56	N901	Beckman Couter	A79388	2	
BV711	CXCR3	1C6/CX3CR	BD Horizon	563156	3	
BV510	CCR7	3D12	BD Horizon	563449	5	
BV650	CD69	FN50	BioLegend	562899	4	
BV786	Ki67	B56	BD	563756	4	Followed the intracellular staining protocol from the eBioscience Foxp3/Transcription Factor Staining kit

Panel #2

Fluorochrome	Marker	Clone	Company	Catalog Number	Volume (uL) per 0.5 - 2 million cells	Note
APC	CD45-Ax647	Custom made, see methods			0.2	Added *in vitro* for animals G45T and HAXR.
PE	CD45-Ax555	Custom made, see methods			0.2	
FITC	CD45-Ax488	Custom made, see methods			0.2	
mCherry	CD45-Ax594	Custom made, see methods			0.2	
Alexa 700	CD45-Alex680	Custom made, see methods			0.2	Added *in vitro* for animals DGA0 and DGF5.
APC-cy7	CD3	SP34-2	BD Pharmingen	557757	4	
APC-cy7	CD14	M5E2	BioLegend	301820	4	
APC-cy7	CD20	L27	BD	335794	4	
PE-cy7	NKG2A	Z199	Beckman Couter	B10246	2	
BV605	CD16	3G8	BD Horizon	563172	2	
PE-CY5.5	CD56	N901	Beckman Couter	A79388	2	
PE-CY5	NKp46	BAB281	Beckman Couter	A66902	4	
Alexa 405 (BV421)	CD49a	TS2/7	Purified Ab: BioLegend Dye: Thermo	Ab: 328302 Dye: A30000	10	Conjugated in-house using NHS ester chemistry.
BV510	CD161	HP-3G10	BioLegend	339922	8	
BV650	CD69	FN50	BioLegend	562899	4	

For CD16 depletion animal (HAXR)

Fluorochrome	Marker	Clone	Company	Catalog Number	Volume (uL) per 0.5 - 2 million cells	Note
VioBlue	CD16	VEP13	Miltenyi Biotec	130-099-080	2	To avoid using the same clone as the CD16-depleting antibody.
